# A Home-Based Balance Exercise Training Program with Intermittent Visual Deprivation for Persons with Chronic Incomplete Spinal Cord Injury: A Pilot Study on Feasibility, Acceptability, and Preliminary Outcomes

**DOI:** 10.3390/s25206320

**Published:** 2025-10-13

**Authors:** Riccardo Bravi, Sara Guarducci, Giulia Panconi, Magdalena Sicher, Lorenzo Mucchi, Giacomo Lucchesi, Gabriele Righi, Giulio Del Popolo, Diego Minciacchi

**Affiliations:** 1 Department of Experimental and Clinical Medicine Affiliation, Physiological Sciences Section, University of Florence, Viale Morgagni 63, 50134 Florence, Italy; giulia.panconi@unifi.it (G.P.); magdalena.sicher@edu.unifi.it (M.S.); diego.minciacchi@unifi.it (D.M.); 2 Department of Information Engineering, University of Florence, Via Di S. Marta 3, 50139 Florence, Italy; sara.guarducci@unifi.it (S.G.); lorenzo.mucchi@unifi.it (L.M.); 3 Spinal Unit, Azienda Ospedaliero-Universitaria Careggi, Largo Piero Palagi 1, 50139 Florence, Italy; lucchesig@aou-careggi.toscana.it (G.L.); righiga@aou-careggi.toscana.it (G.R.); delpopolog@aou-careggi.toscana.it (G.D.P.)

**Keywords:** physical exercise, balance training, postural control, gait, stroboscopic eyewear

## Abstract

Incomplete spinal cord injury (iSCI) results in impaired postural control and walking ability. Visual over-reliance may occur in iSCI individuals to maintain postural control. This can challenge their postural stability in various contexts of daily life activities. The present study assessed the feasibility, acceptability, and preliminary outcomes of balance training with intermittent visual deprivation using stroboscopic glasses on postural control and visual reliance during quiet standing in iSCI individuals. Training impact on walking performance was also evaluated. Seven chronic iSCI individuals participated in a 6-week home-based balance training program, three times weekly, using stroboscopic glasses. Postural and walking abilities were assessed pre- and post-training using a bipedal stance test (BST) and 10 m walking test (10 MWT). BST was performed, with open-eyes (OE) and closed-eyes (CE), on a force plate for three 30 s trials. The center of pressure (CoP) variables included were CoP area (A-CoP) and CoP mean velocity (MV-CoP). Romberg ratios (CE/OE) for two CoP variables were calculated. Duration and speed were measured in 10 MWT. Intervention feasibility was assessed using the feasibility and acceptability questionnaire. Data from able-bodied individuals were recorded and used as references of physiological performance. iSCI individuals were significantly less stable and showed visual over-reliance for postural steadiness compared to controls. Also, their walking ability was impaired. All iSCI individuals completed the training (adherence rate: 84%) and rated it highly feasible. A-CoP and MV-CoP significantly reduced after training in CE condition (*p* = 0.018, respectively) but not in OE condition (*p* > 0.05). The Romberg ratio of A-CoP was significantly lower (*p* = 0.018), but the Romberg ratio of MV-CoP was not (*p* > 0.05). A significant reduction in duration and increase in speed (*p* = 0.018, respectively) in performing the 10 MWT were observed. Preliminary findings from this explorative study indicated that 6-week home-based balance training with intermittent visual deprivation was feasible, acceptable, and had promising potential benefits in improving postural control with a reduction in visual over-reliance in iSCI individuals. The training enhanced also their walking performance.

## 1. Introduction

Globally, it is estimated that over 15 million people worldwide experience a spinal cord injury (SCI), a condition which drastically impacts on health status, quality of life, and life expectancy of the injured person [1]. When SCI occurs, the person faces a sudden and generally everlasting impairment of sensory and motor functioning, of which the magnitude differs based on the lesion’s anatomical level and the number of neural elements involved in the injury [2]. Incomplete lesion of spinal cord is a very common medical condition among people suffering from SCIs of traumatic origin [3]. Following this injury, partially impaired motor and sensory functions below the level of lesion still allow for upright stance and the ability to walk. However, post-lesion balance and walking performances are significantly deteriorated, resulting in a major challenge for most individuals during activities of daily living [4,5,6]. Recovery of balance and gait is considered a top priority for individuals with iSCI [7].

Postural control is the foundation for successful balance and gait performance. The effectiveness of the postural control system depends on the availability and reliability of sensory information from the somatosensory, visual, and vestibular systems during interactions of the body with the constantly changing surrounding environment [8,9,10]. Somatosensory information is suggested to be the main and reliable source of input used for postural steadiness in healthy individuals [5,11,12]. Following spinal cord damage, the relative contribution of the somatosensory system in maintaining balance can be affected due to its reduction and its inaccuracy in providing information on the position of the body in space and the correct perception of limits of stability [13,14]. As a consequence of these deficits, there seems to be an increase in the relative contribution of visual sensory inputs, with them becoming a key source of sensory information for achieving postural stability [5,15,16]. The alteration in reweighting sensory information results from the development of compensatory strategies to maintain balance and can further challenge postural stability in this population [5,15]. As previously reported, 75% of persons with iSCI experience injurious falls while standing and constant loss of balance in the post-rehabilitation period [2,17], which then can decrease social involvement and lead to them developing a fear of falling [2,4,18,19]. Therefore, an innovative approach to restore balance in persons with iSCI would be to reduce over-reliance on visual information and maximize the remaining somatosensory information [5].

Visual deprivation balance training has been shown to be effective in restoring balance and walking abilities in neurological populations that depend on visual information due to somatosensory deficits such as stroke [20,21,22]. Exercising with visual deprivation seems to promote the stimulation of the somatosensory and the vestibular systems, reducing visual dependency [22]. Thus, we speculate that such an approach could benefit also the iSCI population, helping them to restore balance and induce sensory reweighting. However, balance exercise programs using visual restriction have traditionally only been designed with the extreme condition of no vision (blindfolded) [20,21,22]. Given the dependence iSCI persons have on visual feedback to maintain upright posture, the use of an extreme condition such as no-vision can make it very difficult, or even impossible, for these people to perform static and dynamic exercises without adequate supervision and/or support [21,23,24,25]. A training methodology that allows for the possibility to restrict the visual system to some degree between eyes closed and eyes opened could be an appropriate solution for this challenge.

Stroboscopic eyewear is a tool in which liquid crystal technology is employed to make lenses change intermittently between the clear and opaque state, partially blocking the visual information of the wearer (defined as stroboscopic vision) [23,26]. There is initial evidence that the use of stroboscopic glasses when integrated with balance training programs can be effective in restoring impaired postural control and promoting sensory reweighting in populations that over-rely on visual information to maintain balance [25]. According to this, stroboscopic eyewear may be an effective tool for implementation during balance training interventions targeted at restoring balance control and reducing visual feedback over-reliance in persons with iSCI.

The aim of this explorative study was to evaluate the feasibility, acceptability, and preliminary outcomes of a home-based balance exercise training program with stroboscopic glasses on static postural control in a group of persons with iSCI and discover whether or not there is enough suggestion of benefit to warrant further research. Firstly, we assessed if the intervention was feasible and acceptable. Secondly, we tested whether the balance training with intermittent visual deprivation would promote improvements of impaired postural performance and induce sensory reweighting to maintain balance. Finally, we investigated whether the balance exercise program proposed in this study might influence walking performance in iSCI persons.

## 2. Materials and Methods

### 2.1. Participants

A sample of 7 outpatient individuals (5 males, 2 females) with incomplete spinal cord injury were enrolled on the study. The sample size of participants was not determined a priori due to the exploratory nature of the present study, which is based on an innovative experimental intervention never used before in individuals with iSCI. Participants were adults aged from 30 and 57 y with a chronic motor iSCI/D [i.e., American Spinal Injury Association Impairment Scale (AIS) rating of D according to the International Standards for Neurological Classification of Spinal Cord Injury [27,28] of traumatic etiology.

The individuals included in the study were all contacted by phone using the research database of the Spinal Unit at the Florence University Hospital. The following inclusion criteria were adopted to select the sample: 18–65 years of age; incomplete SCI classified as AIS D; chronic SCI lesion [3,29]; the ability to stand independently in an upright position for at least 30 s [30,31]; the ability to walk independently over ground, with or without a gait aid [6,15]; and no participation in any new exercise programs or research interventions during the study period that could have affected the outcome of this study [32,33]. The persons were excluded if they presented: mild to severe spasticity affecting the lower extremity muscles; any significant orthopedic complications, spasms, or contractures; visual impairments; vertigo or vestibular dysfunction; any history of cardiovascular, pulmonary, or metabolic disorders; and a history of seizures, migraines, or light sensitivity [34,35]. The sample’s age range was chosen to minimize the possible influence of old age on visual reliance [36,37,38]. Characteristics of the participants with iSCI are displayed in Table 1. Moreover, 7 able-bodied healthy (ABH) individuals were enrolled and matched to those with iSCI as closely as possible for age, height, weight, and sex. The study was conducted in accordance with the Declaration of Helsinki and approved by the Institutional Review Board of the University of Florence (protocol code: 21088_spe). Informed consent was obtained from all individuals involved in the study.

### 2.2. Study Design

All iSCI participants performed a home-based balance exercise program with stroboscopic eyewear for six weeks and attended two testing sessions (one pre-training and one post-training assessment).

**Figure 1:**
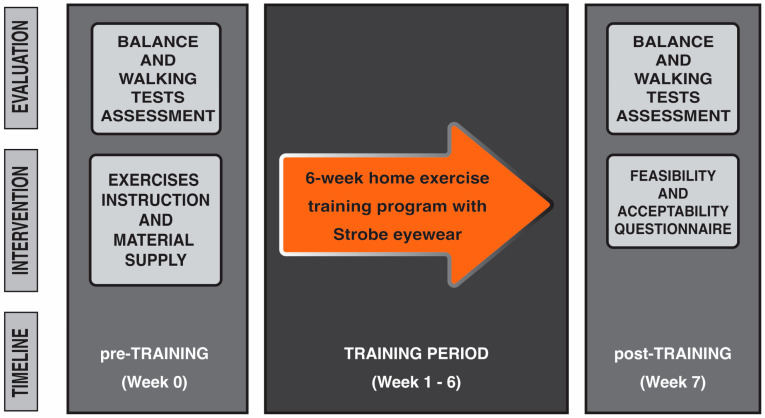
Study paradigm. All persons with iSCI performed a 6-week home-based exercise training program with intermittent visual deprivation using stroboscopic eyewear. One set of pre-training measurements were taken the week before starting the training program and after obtaining the informed consent from the person. On the same day, iSCI persons were instructed on the exercise to perform at home and the usage of stroboscopic eyewear. Each person was retested the week after the completion of training.

**Figure 2:**
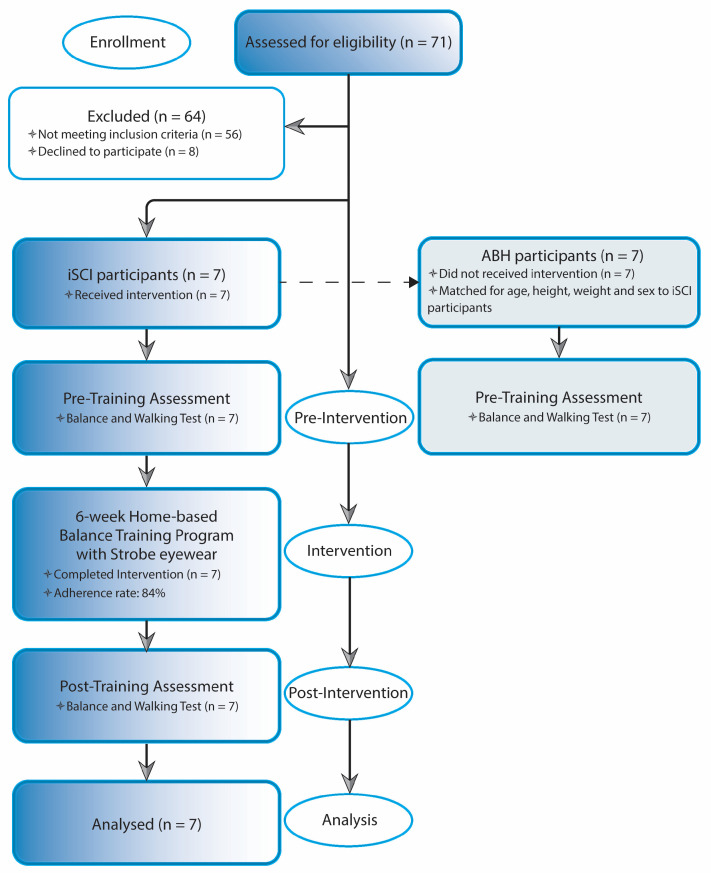
Study Flowchart. The flowchart illustrates the progression of iSCI participants through the stages of the study, beginning with group recruitment and baseline/pre-training assessment, followed by the training program phase, and concluding with the post-training assessment. Final analysis included only iSCI participants who completed both pre- and post-training measures. ABH control participants were also enrolled and underwent baseline assessment to employ their data as reference of physiological performance. Abbreviations: iSCI, incomplete spinal cord injury; ABH, able-bodied healthy.

Each individual who had expressed during the first call the willingness to be considered for research participation made a visit to the Spinal Unit at the Florence University Hospital. At this event they received all information concerning the objectives and requirements of the study and were required to provide informed consent prior to their participation in the study. After that, pre-training measurements for evaluating balance and walking performances were taken. Participants were then instructed how to perform balance exercises. The use of stroboscopic glasses was also explained and tested to ensure safety, feasibility, and effectiveness for home-based exercises. Exercise guidelines containing pictures and written instructions, with an emphasis on safety, were revised and provided in a booklet, as well as video tutorials for home review. The exercise materials including strobe eyewear, foam surface, and guidelines were provided to each participant at this point, and required to return them at the end of the training period. The home-based balance exercise training period started from the following week and lasted for six weeks. Each person was retested for balance and walking performance the week after the completion of training during a post-training visit. At this visit, the person was also asked to complete the feasibility and acceptability questionnaire to obtain information on the usability of, and their satisfaction with, the home-based balance exercise training program with stroboscopic eyewear (Figure 1). Able-bodied healthy individuals underwent the same balance and walking tests assessment once (week 0), and their data were employed as references of physiological balance and walking performance (Figure 2 shows the flow diagram of the study’s progress).

### 2.3. Stroboscopic Eyewear Device

Stroboscopic eyewear is a tool which was first used in the sports field for training purposes more than a decade ago, with the aim of improving the visual, perceptual, and cognitive skills of athletes and consequently athletic performance [23]. Using liquid crystal technology, stroboscopic lenses change intermittently between the clear and opaque state, partially blocking the visual information of the wearer during sports practice [26]. The basic rationale for using stroboscopic glasses during training is that the interruption of visual feedback while practicing may strengthen visual–cognitive processing in order to adapt to the suboptimal information available during the intermittent periods of the opaque state [39]. Results from several studies indicated beneficial effects of specific stroboscopic protocols in general perceptual–cognitive functions and motor skills in healthy athletes [34,40,41,42].

A Senaptec Strobe eyewear device (Senaptec, Beaverton, OR, USA; https://senaptec.com/, accessed on 10 October 2024) was used during the 6-week home-based balance exercise program. The flickering rate of these glasses can be customized through 8 levels, where the clear state is held constant at 100 ms and the opaque state ranges from 67 to 900 ms (5.99–1 Hz cycle). As aforementioned, the present study is the first to use strobe eyewear during a balance exercise program for persons with iSCI. Considering that, the level used for the exercise regimen was selected according to the specifications of Senaptec. Thus, for safety purposes and as a precaution, level 6 was selected [opaque state duration of 471 ms (1.75 Hz)] from the 8 levels as it corresponds to the least stressful stroboscopic viewing condition and is proposed for specifically training balance skills. Finally, binocular viewing mode, i.e., strobing in both lenses, was chosen.

### 2.4. Intervention

All participants performed a structured home-based balance exercise training program 3 times per week for 6 weeks. The exercise program was designed by an interdisciplinary team composed of a physician specialized in the treatment of SCI, a kinesiologist with expertise in postural control and the biomechanics of gait, and a physiotherapist with several years of experience in neurorehabilitation. The balance exercise program was divided into two training periods (first period: weeks 1–3; second period: weeks 4–6) with an increasing difficulty of balance exercises through the two phases in order to provide optimal exercise progression and preserve motivation throughout the course of the program [43,44]. From the second period a foam surface was integrated into some exercises to induce more challenging balance exercises [24,45]. Each training session was unsupervised and carried out at the home of each participant. Each lasted about 45 min and consisted of a warm-up, balance exercises, and a cool-down. The balance exercises phase of every training session comprised the execution of six exercises, each of which was chosen from previous training programs to improve balance and walking abilities in subjects with impaired postural control [31,44,46,47,48,49]. Specifically, balance exercises we proposed, in line with previous literature [44,50,51,52], were classified as stable, weight-shifting, and mobile exercises (Table 2). Every exercise was tailored to each participant’s abilities [53]. In this sense, participants could advance through three levels of difficulty for each exercise, with a decrease in the amount of support provided for safety. The support for safety used by each participant was adapted to a personal home setup by using available equipment (e.g., secured, stable furniture, or house wall). At Level 1 (easy), participants used both hands for full support, ensuring maximum stability and minimizing the risk of losing balance. At Level 2 (moderate), the amount of support was reduced, with participants using only one hand or lightly touching the support with their fingertips, requiring greater control and coordination. At Level 3 (difficult), exercises were performed with no support, relying entirely on participants’ ability to maintain control throughout the exercises. At Level 3, participants were encouraged to only use the support when they needed to. Depending on the specific exercise, each exercise was required to be performed for approximately three to five minutes. All participants were instructed to wear the stroboscopic eyewear in the active mode during the execution of the exercise, except for at rest breaks. Rest breaks between exercises were taken as needed. Please refer to Appendix A for a detailed description of the exercises adopted in two periods of the training program.

All balance exercises were demonstrated by an exercise supervisor and every participant was made to try them first without and then with stroboscopic eyewear in active mode during the pre-training assessment day (after collecting balance and gait measurements). Also, an exercise booklet was provided as a guide, containing a series of pictures and written instructions on how to perform each exercise with proper form and technique along with a video tutorial for home review [53,54]. A weekly exercise record was registered by each participant using an online questionnaire on the virtual platform of Google Moduli. Furthermore, the exercise supervisor contacted each participant weekly by phone to supervise any changes in the training program, to monitor the program’s progression, to answer questions, and provide indications and advice to perform exercises, as well as to encourage adherence [53]. Finally, participants were asked to immediately contact the exercise supervisor if there were any possible adverse events during the home-based balance exercise training program, and to report them as well in the online questionnaire [53].

### 2.5. Setup and Evaluation of Outcomes (Data Collection—Measurements)

#### 2.5.1. Feasibility and Acceptability

Information concerning the usability and satisfaction of participants undertaking the home-based balance exercise training program with stroboscopic eyewear was obtained by having them complete the feasibility and acceptability questionnaire, adapted from Hansen et al. (2022) [55]. Specifically, during the post-training visit, participants were requested to fill out the questionnaire and rate the feasibility and acceptability of the exercise training program. The feasibility and acceptability questionnaire consisted of 6 items arranged on a Likert scale. Four items were related to the assigned exercise training program and two items were related to exercise training program guidance (see Table 3 to see the items). The Likert scale used was scored as follows: 1 (strongly disagree), 2 (disagree), 3 (moderately agree), 4 (agree), and 5 (strongly agree) [55]. A median score of ≥3.0 was adopted as a criterion to indicate that the home-based balance exercise training program with stroboscopic eyewear was perceived as acceptable [55]. In order to assess the feasibility and the acceptability of the home-based balance exercise training program, adherence rate was also calculated. Adherence rate was calculated as the percentage of total completed training sessions (of all participants) out of total prescribed sessions, where 100% adherence meant the completion of 126 sessions (3 sessions/week for 6 weeks for seven participants) [55,56]. When participants missed any exercise sessions during one week, they was given the following week to complete the make-up sessions.

#### 2.5.2. Primary Outcome: Balance Performance

Static balance performance was evaluated using a monoaxial force platform (PK 252, TecnoBody S.r.l., Italy), consisting of a surface with a 55 cm diameter, a 20 Hz sampling rate, and a sensitivity of 0.1 [57]. Each participant was required to stand barefoot on the balance surface, in an upright static condition, double-leg stance, with arms by their sides, with both heels lined up, and was instructed to look straight ahead. According to the platform specifications, the position of their feet on the force platform was standardized between pre- and post-training tests via a silk-screened V-shaped frame on the surface of the system, with each foot abducted 15° with respect to the sagittal axis and the heels separated by 2 cm with respect to the horizontal axis. Balance performance was tested in two visual conditions: open eyes (OE) and closed eyes (CE). Three trials per each visual condition were performed. During the bipedal stance test, the participant was instructed to stand on the balance surface as still as possible for 30 s. The instant positions of the center of pressure (CoP) on the ground were recorded during the trials. Based on previous reports [5,15,58], the CoP area (A, mm^2^), the ellipse area that encloses 95% of the points on the CoP path, and the CoP mean velocity (MV, mm/s) were chosen to examine balance performance. CoP mean velocity was calculated by dividing the CoP path length (the sum of the displacements of CoP on the force-measuring platform) for the duration of the recording. Romberg ratios (CE/OE performances) for both CoP variables were also calculated to assess the contribution of sensory information, specifically the influence of visual inputs on regulating postural steadiness [15,25]. Lower CoP area and CoP mean velocity values were interpreted as reflecting better postural control [5,58,59], while lower Romberg ratios scores was interpreted as indicating lower reliance on visual information in maintaining standing balance [15]. Lastly, during testing an operator always stood behind the participant to prevent falls.

#### 2.5.3. Secondary Outcome: Walking Performance

The ten meters walking test (10 MWT) is a method validated to assess gait ability in individuals with iSCI [60,61,62] with an excellent test–retest reliability (r = 0.983) and inter-rater reliability (r = 0.974) [60]. It measures the time that it takes a participant to walk 10 m in a straight line [63,64]. The test was performed on a flat, smooth, non-slippery surface, with no disturbing factors [65]. The participants were instructed to walk as fast, but safely, as possible [64,65,66], using a gait aid if necessary. As previously described [62,63,66], the 10 MWT was performed with a “flying start”, with participant walking 2 m before the time was recorded and 2 m after to avoid bias because of acceleration and deceleration. The time taken to walk the 10 m distance was measured using a stopwatch. Each of the three trials performed by the participants started with the word “Go” from the operator. Timing started and stopped when the participant’s lead leg broke the plane of the markers placed on the floor at the beginning and at the end of the 10 m path, respectively. Duration and speed were used as variables to assess walking performance. Speed was calculated by dividing the 10 m walking distance with the walking time (m/s).

### 2.6. Statistical Analysis

The sample size of iSCI individuals used in this investigation was in line with previous studies on iSCIs in which the effects of physical-related interventions to improve postural control were evaluated [32,67]. A post hoc power analysis was conducted using G*Power 3.1 (Heinrich Heine University, Düsseldorf, Germany), based on the Wilcoxon signed-rank test (matched pairs). The expected effect size (d = 1.98) was derived from a previous randomized controlled study that investigated the effects of balance training using stroboscopic eyewear in a population that was shown to over-rely on visual feedback during postural control. The effect size was estimated utilizing the within-group variance of CoP mediolateral velocity change scores between pre- and post-intervention measurements in CE condition (pre-intervention: mean = 24 mm/s; SD = 5.9 mm/s; post-intervention: mean = 13 mm/s; SD = 5.1 mm/s) [25]. With α = 0.05, two-tailed, and a total sample size of 7, the achieved statistical power (1 − β) was 0.98.

The Shapiro–Wilk test (*p* < 0.05) [68,69] as well as an inspection of the skewness and kurtosis measures [70,71,72] evidenced that the data were not normally distributed. Due to this, the equality of variance for the group of individuals with iSCI and the control group of ABH individuals was verified using a non-parametric Levene’s test [73,74]. The Mann–Whitney U test was used to compare demographics (age, height, and weight, respectively) as well as balance and walking performance between the iSCI and ABH groups at baseline (T0). The chi-squared test was also used to test for sex differences between the two groups. Moreover, the Wilcoxon signed-rank test was used to assess the effect of the balance training program with active intermittent visual deprivation on the balance and walking performance in the iSCI group. Significance level was set to alpha = 0.05. Effect size was estimated using Pearson’s correlation, r. According to Cohen’s recommendations for the interpretation of effect size magnitude, >0.1, >0.3, and >0.5 were considered small, medium and large effects, respectively [75,76,77]. Finally, with the aim of missing important findings on the effects of a new balance approach as it could be investigated in detail in future studies, we preferred to follow a policy of not making adjustments for multiple comparisons to reduce any errors of in-terpretation from the obtained preliminary observations of a new method to improve balance and gait performances in iSCI [78,79,80].

## 3. Results

### 3.1. Feasibility and Acceptability

All of the participants with iSCI successfully completed the six-week home-based balance exercise training program. Feasibility and acceptability scores of the home-based balance exercise training program with active intermittent visual deprivation are presented in Table 3. Overall, all six items of the feasibility and acceptability questionnaire reached a median score of ≥4.0, indicating that participants perceived home-based balance exercise training intervention as feasible and acceptable. In addition, out of the total 126 sessions, 106 were completed (adherence rate: 84%). Reasons reported by participants for not completing the sessions included lack of time, sickness, and fatigue. No adverse events related to the interventions occurred or were reported.

### 3.2. Comparison of Demographics Between iSCI and ABH Individuals

Table 4 shows the demographics of the ABH and iSCI groups. No significant differences between the two groups were found for age, height, weight, and sex.

### 3.3. Balance Performance

A Mann–Whitney U test was conducted to compare the balance performance between the ABH and iSCI groups at baseline T0. As expected, the test showed significantly larger scores in the iSCI group compared to the ABH controls in A-CoP and MV-CoP for both OE and CE conditions, indicating poorer balance control for the iSCI group in performing the bipedal stance test. Also, the Romberg ratio of A-CoP, but not the Romberg ratio of MV-CoP, was found to be significantly higher in the iSCI group compared to the ABH controls, suggesting an increased reliance on visual inputs in iSCI individuals for postural steadiness.

**Figure 3:**
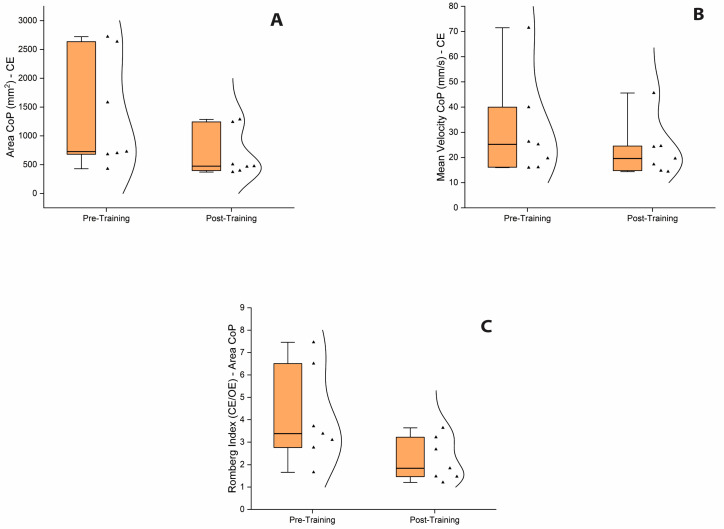
Box and whisker plots of balance performance during bipedal stance test. (**A**). Box and whisker plot showing the significant reduction in A-CoP scores from pre-training to post-training assessment in CE condition. (**B**). Box and whisker plot showing the significant reduction in MV-CoP scores from pre-training to post-training assessment in CE condition. (**C**). Box and whisker plot showing the significant reduction in the Romberg ratio index of A-CoP from pre-training to post-training assessment. Inside the boxes are the medians (thick lines) and spreads of 50% of central values (25th to median and median to 75th). Outside the boxes are the upper and lower limits of the range of values. Kernel smooth distribution provides an estimate of the probability density of the data at different values. Abbreviations: OE, open eyes; CE, closed eyes; CoP, center of pressure.

Moreover, a Wilcoxon signed-rank test was used to assess the effect of the balance training program in the iSCI group. The test revealed a significant reduction in A-CoP and MV-CoP scores from pre-training to post-training assessment when bipedal stance was performed in the CE condition, which may indicate a post-training improvement in balance control for iSCI when vision was occluded (Figure 3A,B). Yet, no significant pre- and post-training differences in A-CoP and MV-CoP scores were revealed when bipedal stance was performed in the OE condition. Finally, the Romberg ratio of A-CoP, but not the Romberg ratio of MV-CoP, was found to be significantly reduced from the pre-training to post-training assessment, indicating that the balance exercise program with intermittent visual deprivation might have promoted a reduction in the over-reliance on visual information for maintaining stance stability in the iSCI group (Figure 3C). All statistical details are presented in Table 5.

### 3.4. Walking Performance

**Figure 4:**
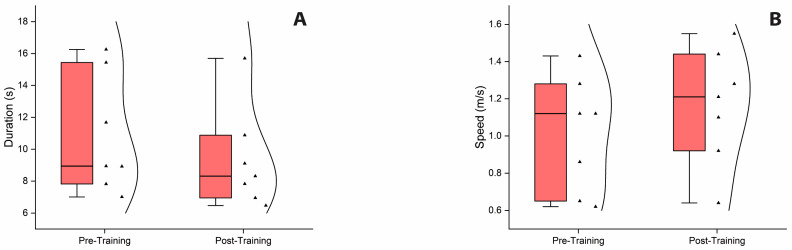
Box and whisker plots of ten meters walking performance. (**A**). Box and whisker plot showing a significant reduction in duration in performing 10 MWT from pre-training to post-training assessment. (**B**). Box and whisker plot showing a significant increase in speed from pre-training to post-training assessment. Inside the boxes are the medians (thick lines) and spreads of 50% of central values (25th to median and median to 75th). Outside the boxes are the upper and lower limits of the range of values. Kernel smooth distribution provides an estimate of the probability density of the data at different values. Abbreviation: 10 MWT, ten meters walking test.

The Mann–Whitney U test showed significantly higher duration scores and lower speed scores in the iSCI group compared to the ABH controls in performing 10 MWT at baseline T0, indicating that walking abilities, similar to balance control, were impaired in the iSCI group. Furthermore, the Wilcoxon signed-rank test revealed a significant reduction in duration and an increase in speed from pre-training to post-training assessment, indicating an improvement in walking performance for the iSCI group after the training period (Figure 4A,B). All statistical details are presented in Table 6.

## 4. Discussion

This study aimed to evaluate the feasibility, acceptability, and preliminary outcomes of a home-based balance exercise training program with stroboscopic glasses which was looking to influence postural control and induce sensory reweighting in persons with iSCI. The potential benefits of balance training on walking performance were also assessed. Bipedal stance and ten meters walking tests were used to evaluate potential improvements in balance control and walking abilities.

### 4.1. Feasibility, Acceptability and Adherence

At the end of training, persons with iSCI rated the exercise program to be highly feasible and acceptable. Specifically, participants strongly agreed that the proposed balance exercises with stroboscopic eyewear were worth their time. The exercises were also considered easy to perform, with a median score of 4 out of 5. Even though exercises were unsupervised, as expected for a home-based training program like in our study, the adherence rate was 84% with complete participant retention. This is considerably high compared to adherence rates reported in other unsupervised exercise programs for iSCI persons [81,82].

The positive program adherence might have been influenced by several factors regarding the nature of the exercise program and participants’ enjoyment towards it. The training program was conducted at each participant’s home, on self-selected days and at self-selected times every week, using provided equipment. The advantages of this home-based program were as follows: low cost, the convenience of exercising in a comfortable environment, saving on travel time and transport fees, and elimination of possible transport difficulties. It has been shown that the most common causes of non-adherence to exercise intervention programs for iSCI persons are problems with transportation, long distance to rehabilitation centers, and high cost of physiotherapeutic service [83,84]. Furthermore, it is worth mentioning that in our results participants agreed that the balance exercises using stroboscopic eyewear were fun to do. This could have contributed to the high adherence rate besides general benefits of the training program. The reaction to the intervention, that is, whether the participants enjoyed it or not, was shown to be a factor promoting regular physical activity and reducing dropouts [85,86]. It should be mentioned that our program was designed with a progression of balance exercises difficulty throughout the two phases and lasted for only 6 weeks, which is a relatively short period of training. This might encourage participants to maintain their motivation and engagement in the program and hence, increase the adherence rate [55,87].

### 4.2. Potential Benefits on Postural Control

Postural control is represented by three commonly studied aspects: stability performance, control demand, and postural regulations [5,88]. Stability performance indicates the ability to maintain balance within a stability limit, that is, the space boundaries in which the body can maintain its position without changing the base of support [89]. Control demand is described as the attentional resources used to maintain stability [5,90]. It is suggested that CoP time-domain area measures, which estimate the displacement of the CoP, are indicators of stability performance, while CoP time-domain velocity measures are related to control demand [88]. A decrease in stability performance (i.e., increased CoP displacement) accompanied by increased control demand (i.e., increased CoP velocity) is often associated with deterioration of postural control [5,90].

The present study examined the CoP area and CoP mean velocity measures to assess postural control performance in iSCI persons. Romberg ratios (CE/OE performances) for both COP variables were calculated to quantify the influence of visual inputs on postural steadiness which could be related to sensory reweighting strategy post-injury [15,45].

The comparison results in the present study showed larger CoP area and increased CoP mean velocity measures in iSCI in both OE and CE conditions, indicating reduced balance steadiness in individuals with iSCI compared to able-bodied healthy controls. In addition, the Romberg ratio of CoP area in the iSCI group was found to be higher than that of healthy controls, suggesting an increased reliance on visual inputs among individuals with iSCI to maintain balance control. This is in line with previous literature investigating how spinal cord injury affects postural control in iSCI populations. According to these studies, the reduction and the decreased accuracy of somatosensory information post-injury might result in compensation strategies regarding postural control, in which the relative contribution of visual sensory inputs is increased in order to achieve balance [5,15,16].

While this increased reliance on visual feedback can produce functional benefits in maintaining balance, the residual impaired somatosensory information might lead to imbalance and higher risk of falling due to the insufficiency and inaccuracy of required sensory inputs under multitask conditions (e.g., reading while walking) or difficult sensory contexts during daily life activities (e.g., dark conditions or environmental hazards such as unexpected obstacles) [91,92]. In this sense, the balance training employed in the present study has made, for the first time, an approach to improve balance control and reduce visual reliance in persons with iSCI using exercises with intermittent visual deprivation.

Results may indicate an improvement of balance control in individuals with iSCI according to the reduction in CoP area and CoP mean velocity biomarkers in the post-training assessment. Interestingly, the effect of balance training was only found when participants performed the bipedal stance test in CE condition, in which vision was occluded. These results, accompanied by lower Romberg ratio scores for CoP area, may provide preliminary indications for the potential benefits of stroboscopic balance training in improving stance stability and reducing over-reliance on visual information. The iSCI group in CE condition might have been able to use other sensory information, such as somatosensory and/or vestibular information, to maintain their static postural control successfully [12]. In our study, the balance test was performed bipedally on a stationary, horizontal surface that provides a stable frame of reference for postural control, a condition in which the contribution of vestibular information was shown to be not critical for maintaining an upright stance [93,94,95,96]. Therefore, it is possible that the central nervous system more effectively used somatosensory information through sensory reweighting in the absence of visual feedback.

We speculate that during the training program, balance exercises with stroboscopic glasses might have triggered sensory reweighting, an adaptive compensation strategy which occurs when the nervous system dynamically weighs degrees of reliance on the sensory information and chooses to use more of reliable sensory modalities and less of unreliable modalities for postural control [11,97,98,99]. Such a training method, in which visual information is reduced by intermittent visual occlusions, possibly led the central nervous system to adapt to contexts of limited visual feedback by decreasing the reliance on it and employing more somatosensory information to control balance [100]. It could explain for the post-training improvements on postural control and a reduction in visual reliance in iSCI persons. A recent study by Lee and coworkers (2022) found similar effects for a 4-week balance training program with stroboscopic glasses on postural control in individuals with chronic ankle instability, a population that has also been shown to over-rely on visual feedback during postural control due to impaired somatosensory function. In this study, authors showed that the training successfully enhanced postural control and decreased visual reliance in this population [25].

However, in our study, no significant effect of stroboscopic balance training was found when persons with iSCI performed the bipedal stance test when vision was available. One possible explanation could be that under a different context (OE), roles of visual and somatosensory information were affected and individuals increased their reliance on visual inputs which now became reliable sensory sources. It has already been shown in the literature that during a quiet stance on a firm platform when vision is available, subjects reduce reliance on somatosensory inputs and increase reliance on visual information [45,101]. The characteristics of the bipedal stance test, for this reason, might not show an evident change in postural control performance after the training. Future studies could evaluate the effects of stroboscopic training in different conditions in which the contribution of somatosensory feedback is considered to be more important for postural control when visual information is available.

### 4.3. Improvement in Walking Performance

The recovery of walking is a very important goal in persons with iSCI [102]. Our study, consistent with previous literature, showed that walking was impaired in the iSCI group, with iSCI persons performing 10 MWT with higher duration and lower speed in comparison to able-bodied healthy controls [32,59]. The post-training evaluation showed improvements in both duration and walking speed, suggesting a possible beneficial effect of the six-week home-based balance exercise training program on enhancing walking performance in iSCI persons.

Previous studies have observed the link between balance and gait, with balance seen to be a key factor for walking recovery in iSCI populations. Scivoletto and colleagues showed that balance, strength, spasticity, and age were correlated with walking performance in chronic iSCI persons, with higher balance ability related to higher speed in performing 10 MWT [63]. Similarly, in a multicenter clinical trial study by Ditunno et al. (2007), the authors found correlations between walking speed and functional capacity measures of balance in acute iSCI persons [103]. Finally, Tamburella et al. (2013) showed that a program with conventional balance and walking exercises associated with visual biofeedback task-specific balance training promoted the recovery of balance and gait abilities in a group of chronic iSCI persons, and that enhancement in balance preceded the improvement in gait performance [32]. Therefore, in our study we speculate that improvements of postural control following balance training periods might have promoted walking ability in iSCI persons. However, bear in mind that among factors influencing walking performance, greater muscle strength, specifically of the proximal muscles in the lower limbs, was also observed as an important determinant for higher performance in short- and long-distance walking tests in iSCI persons [63]. The fact that whether or not the balance training we designed had improved muscle strength which, in turn, positively influenced walking ability in iSCI remains uncertain as our study did not quantify changes in muscle strength pre- and post-training. The definite mechanism of how balance training with intermittent visual deprivation using stroboscopic eyewear could affect gait performance cannot be concluded within the current scope of this study. It is suggested that further studies on the effect of balance training interventions using stroboscopic eyewear on gait should evaluate the modulation of strength level in participants using the lower-extremity manual muscle test.

Altogether, the home-based balance exercise training with stroboscopic eyewear might have promoted some potential benefits for improving postural control and gait in the iSCI population. This methodology using stroboscopic eyewear enables the possibility to perturb the visual system to any degree between eyes closed and eyes opened. It could be considered an advantage compared to visual-deprivation balance exercise programs where perturbations of visual input are limited to the use of the extreme condition of no vision [24]. It could allow the highly visual dependent iSCI population to perform static and dynamic exercises that otherwise would be very difficult, or even impossible, without supervision and/or support [23,24,25]. Finally, since stroboscopic eyewear technology allows one to modulate the duration of the opaque-state phase, one can use it to tailor visual perturbation based on postural skills and visual dependence of a single person and thus favor the progression of visual deprivation throughout the training as abilities improve [104].

### 4.4. Limitations and Future Directions

Several limitations concerning the current preliminary study should be addressed. Firstly, the number of participants remains relatively small. With a small sample size, our findings might be influenced by individual variability of participants, especially when extreme outcomes from a few individuals could bias the results. Therefore, pre- and post-training results from the Wilcoxon signed-rank test might not accurately represent the true median difference in the studied group. Secondly, the chosen statistical approach for this exploratory study did not account for adjustments for multiple comparisons, which increased the risk of type I error. Thirdly, the study exclusively enrolled individuals with chronic iSCI, meaning that the observed outcomes solely reflect the response of this specific subgroup to the stroboscopic balance training intervention. As such, the findings cannot be generalized to other iSCI populations, such as those in acute or subacute stages of recovery. To enhance generalizability and confirm our preliminary observations, future investigations should aim to recruit larger and more diverse groups, including participants across different phases of injury and recovery. This could be achieved through multicenter collaborations to increase participant availability, combined with stratified recruitment strategies to ensure balanced representation of individuals across the acute, subacute, and chronic stages of iSCI.

Fourth, only one experimental group was established and no comparison with an active iSCI control group undergoing a conventional balance training program was planned for this study. Thus, despite the benefits obtained using this innovative training modality, the employed single-arm design cannot allow for baseline comparison, and hence, renders it unable to disentangle between the effects of other confounding factors (e.g., increased lower limb muscle strength [105]) and the real contribution of intermittent visual deprivation provided by stroboscopic eyewear in promoting the recovery of postural control and sensory reweighting in iSCI. To address this limitation, future studies should adopt randomized-controlled designs with active control groups receiving conventional balance training exercises. This would allow for a comparison between standard training programs and the stroboscopic balance intervention, thereby isolating the specific effects of intermittent visual deprivation.

Another limitation of this study lies in the evaluation of the effect of balance training for only a short-term period. Therefore, an important direction for future research is to conduct studies that include longer-term exercise programs, possibly with interim evaluations to ascertain performance progress over time. In addition, no follow-up performance was assessed after the post-training test and this should be considered an additional drawback. The matter of long-term effects of stroboscopic balance training in iSCI is a future area of investigation.

Moreover, though the training program was designed with a progression of difficulty to keep the program challenging motivation, the challenge level of exercises for each participant after every training session was not rated. A more customized training program where exercise challenge is constantly rated and subsequently adjusted to each individual’s abilities is considered advisable to optimize challenge and enhance improvement throughout the program [47]. Additionally, as in most unsupervised home-based exercise programs, participant adherence in terms of frequency was limited to monitoring by self-report, which might have caused inflated adherence training rates. Indeed, self-report responses may reflect what the person considered as the desired response, rather than what they actually did, giving a false positive result of adherence [106]. To mitigate this limitation in future studies, more objective adherence monitoring strategies should be considered. One promising approach is the use of wearable technologies (e.g., sensors), which are increasingly recognized as effective tools for capturing motion data and tracking home-based physical activity interventions [107,108,109,110]. Furthermore, researchers could use performance metrics collected by the sensors to dynamically adjust exercise difficulty, ensuring a personalized and progressively challenging training program.

Finally, although not included in the present study, estimating the effort produced during quiet standing could offer additional insights into the physiological demands of balance control. Therefore, it is suggested to quantify the effort in terms of the mechanical work required to maintain balance in order to better characterize the efficiency of postural control mechanisms in iSCI persons and the degree of neuromuscular engagement elicited by the stroboscopic training program.

## 5. Conclusions

The present pilot study explored the use of stroboscopic eyewear in a balance exercise program for individuals with chronic incomplete spinal cord injury. Our results suggested that a 6-week home-based balance exercise program with intermittent visual deprivation is a highly feasible and acceptable balance training modality. The training program seems to have the potential to enhance postural control and decrease visual reliance when maintaining stance position. Although preliminary, our findings appear promising and could motivate future trials to integrate stroboscopic eyewear in balance exercise training to improve postural control and reduce over-reliance on visual feedback in this population.

## Figures and Tables

**Table 1:** Demographic information of participants with incomplete spinal cord injury.

Participant	Age (Years)	Sex	Height (cm)	Weight (kg)	Etiology	Level of Lesion	AIS Classification	Time Elapsed Since Injury (Months)	Gait Aid
1	54	Female	166	55	T	L2	D	53	Crutch
2	48	Female	173	69	T	C4-C5	D	9	/
3	57	Male	181	94	T	C3-C4	D	18	/
4	47	Male	180	86	T	C3-C4	D	83	/
5	30	Male	175	70	T	C4-C5	D	8	/
6	53	Male	179	84	T	C7-D1	D	33	/
7	38	Male	182	88	T	D12	D	48	Cane

Abbreviations: T, traumatic; AIS, American Spinal Injury Association Impairment Scale.

**Table 2:** Categories of exercises and their examples from the home-based balance training program.

Category			Exercise Examples
**Standing**			dual limb and single limb stance *, unilateral balance with limb movement
**Weight-shifting**			weight shift *, heels ups/toes ups, sit to stand to sit, half squat and squat *, alternating step-ups, lunges
**Walking**			walking, side stepping, tandem walking, turning

* Stable surface progressing to foam surface.

**Table 3:** Feasibility and acceptability of the home-based balance exercise program with stroboscopic eyewear.

Questions	Q1	Q2 (Median)	Q3
1. I found the exercises fun to do	3.5	**4**	5
2. I was able to do the exercise program without difficulty	4	**4**	4
3. The exercises were worth my time to do	4.5	**5**	5
4. The exercises were easy to perform	3	**4**	4.5
5. The instructions on how to perform the exercises and the stroboscopic eyewear during the exercise were clear	4.5	**5**	5
6. I received the guidance I needed from the exercise supervisor	5	**5**	5

Data presented as median (Q1–Q3). Q1 = percentile 25; Q3 = percentile 75.

**Table 4:** Demographic information of participants in the study.

	iSCI Group (n = 7)	ABH Group (n = 7)	p Value
Age (years) ^§^	46.7 (9.60), 30, 57	45.9 (5.52), 35, 51	0.608
Height (cm) ^§^	176.6 (5.68), 166, 182	177.3 (6.97), 169, 190	0.797
Weight (kg) ^§^	78.0 (13.72), 55, 94	68.4 (8.50), 58, 82	0.124
Sex (Males/Females) ^$^	5/2	5/2	1.00

Data presented as means (standard deviations), min, max. ^§^ and ^$^: Mann–Whitney U and chi-square test, respectively. Abbreviations: iSCI, incomplete spinal cord injury; ABH, able-bodied healthy.

**Table 5:** Balance performance and visual contribution during bipedal stance test.

	**CoP** **Parameters**	**ABH**	**iSCI**	**iSCI**	**iSCI vs. ABH**	**iSCI**
		**T0**	**T0**	**T1**	**T0** (U, *P*, r)	**T1 vs. T0** (Z, *P*, r)
**Condition**						
OE	Area	68 (55–86, 44, 148)	241 (160–829, 104, 971)	350 (194–420, 115, 702)	1.0, 0.001, 0.80	−0.169, 0.866, -
	Mean Velocity	9 (6–9, 6, 9)	14 (11–33, 8, 36)	13 (12–17, 11, 29)	4.0, 0.007, 0.70	−1.014, 0.310, -
CE	Area	102 (89–124, 67,127)	727 (681–2636, 428, 2722)	474 (397–1242, 372, 1286)	0.0, 0.001, 0.84	−2.366, 0.018, 0.89
	Mean Velocity	12 (8–14, 8, 15)	25 (16–40, 16, 71)	20 (15–25, 14, 46)	0.0, 0.001, 0.84	−2.366, 0.018, 0.89
**Romberg** **Index** (CE/OE)	Area	1.63 (1.29–1.85, 1.01, 1.99)	3.38 (2.76–6.51, 1.66, 7.46)	1.84 (1.47–3.22, 1.21, 3.64)	3.0, 0.004, 0.73	−2.366, 0.018, 0.89
	Mean Velocity	1.37 (1.31–1.56, 1.06, 1.80)	1.82 (1.22–1.96, 1.21, 1.98)	1.43 (1.40–1.64, 1.19, 1.73)	18.0, 0.456, -	−1.521, 0.128, -

Abbreviations: T0: baseline/pre-training timepoint; T1: post-training timepoint (after six weeks); OE, open eyes; CE, closed eyes; CoP, center of pressure; iSCI, incomplete spinal cord injury; ABH, able-bodied healthy. Data presented as median (percentile 25–percentile 75, min, max). *n* (iSCI) = 7; *n* (ABH) = 7. Significance level was set to alpha = 0.05. r: estimation of the effect size with Pearson’s correlation coefficient.

**Table 6:** Ten meters walking performance.

**Walking** **Parameters**	**ABH**	**iSCI**	**iSCI**	**iSCI vs. ABH**	**iSCI**
	**T0**	**T0**	**T1**	**T0** (U, *P*, r)	**T1 vs. T0** (Z, *P*, r)
Duration	5.09 (4.91–5.69, 4.90, 5.91)	8.94 (7.82–15.44, 7.01, 16.26)	8.31 (6.95–10.88, 6.47, 15.70)	0.0, 0.001, 0.84	−2.366, 0.018, 0.89
Speed	1.97 (1.76–2.04, 1.69, 2.05)	1.12 (0.65–1.28, 0.62, 1.43)	1.21 (0.92–1.44, 0.64, 1.55)	0.0, 0.001, 0.84	2.366, 0.018, 0.89

Abbreviations: T0: baseline/pre-training timepoint; T1: post-training timepoint (after six weeks); iSCI, incomplete spinal cord injury; ABH, able-bodied healthy. Data presented as median (percentile 25–percentile 75, min, max). iSCI *n* = 7 and ABH *n* = 7, respectively. Significance level was set to alpha = 0.050. r: estimation of the effect size with Pearson’s correlation coefficient.

## Data Availability

The raw data supporting the conclusions of this article will be made available by the authors on request.

## Abbreviations

The following abbreviations are used in this manuscript:

SCI	Spinal cord injury
iSCI	Incomplete spinal cord injury
ABH	Able-bodied healthy individuals
OE	Open eyes
CE	Closed eyes
CoP	Center of pressure
A	Area
MV	Mean velocity
10 MWT	Ten meters walking test

## Appendix A. Exercises Adopted During the Training Program

The six exercises for week 1–3 were performed on a firm surface and included:

1. Dual Limb and Single Limb Static Standing:

Starting from standing position with feet shoulder-width apart, focusing on maintaining balance, participants held the position on both legs for 30 s. Next, they lifted one leg off the ground and held it in place, maintaining balance on the supporting leg. After holding the position for 30 s, they slowly lowered the leg and repeated the exercise on the other leg. Participants performed repetitions for 5 min.

2. Unilateral Balance with Limb Movement:

Starting from a standing position with feet together, participants lifted one leg laterally and returned to the starting position. Next, they lifted the leg forward, swung it backward in a controlled motion, and returned to the starting position. Finally, they performed a circular movement, lifting the leg forward with knee bent at 90°, moving it laterally, then extending it backward before returning to the starting position. Participants performed repetitions for 2 and a half minutes per leg.

3. Weight Shifts:

Starting from a standing position with feet shoulder-width apart, participants gently shifted their body weight forward and backward, and then side to side. The movements required maintaining balance without moving the feet, focusing on controlled transitions between positions. Each shift was held for approximately three seconds before returning to the center. Participants performed repetitions for 5 min.

4. Half Squat:

From a standing position with feet shoulder-width apart, participants gently pushed their hips back while bending their knees forward, lowering into a squat approximately halfway between a standing and full squat position, distributing the weight equally on both feet. The position was held for about five seconds before returning to a standing posture, ensuring the feet remained stationary. Participants performed repetitions for 3 min.

5. Sit to Stand to Sit:

From a seated position on chair, with feet staying flat on the floor, participants leaned their trunk to shift weight forward and stood up with control until their legs were completely straight. After achieving a stable standing position, they slowly lowered themselves back into the chair without dropping abruptly. Participants performed repetitions for 3 min.

6. Multi-Path Balance Walk:

Participants walked as steadily as possible, incorporating patterns such as forward and backward walking over several meters, lateral steps to the left and right, and tandem walking, where one foot is placed directly in front of the other with the heel touching the toe. They also navigated small obstacles, turning as necessary. Participants performed these walking patterns for 5 min.

During weeks 4–6, the exercises were designed to progressively increase the difficulty of the balance training program. The most significant change in this phase was the introduction of an unstable surface in some exercises, building upon the movements practiced in the previous weeks. These exercises, while familiar, now presented greater challenge to balance and stability.

The 6 exercises for week 4–6 included:

1. Dual Limb and Single Limb Static Standing on Foam Surface:

This exercise was similar to the static standing exercise from weeks 1–3 but was performed on a foam pad to increase the challenge. Starting from standing position with feet shoulder-width apart, focusing on maintaining balance, participants held the position on both legs for 30 s. Next, they lifted one leg off the ground and held it in place, maintaining balance on the supporting leg. After holding the position for 30 s, they slowly lowered the leg and repeated the exercise on the other leg. Participants performed repetitions for 5 min, keeping their balance on the foam pad.

2. Weight Shifts on Foam Surface and Heels ups/Toes ups:

Starting from a standing position with feet shoulder-width apart on a foam pad, participants gently shifted their body weight forward and backward, then side to side. Afterward, without using the foam pad, they lifted the heels, rising onto their tiptoes and then lowered back down. Finally, they raised their toes, standing on their heels. Each shift was held for approximately three seconds before returning to the center. Participants performed repetitions for 5 min.

3. Squat on Foam Surface:

From a standing position with feet shoulder-width apart on a foam pad, participants gently pushed their hips back while bending their knees forward, lowering their body toward a sitting position, distributing the weight equally on both feet. The goal was to squat until the thighs were parallel to the ground or as close as possible while maintaining proper posture and balance. This position was held for about five seconds before returning to a standing posture, ensuring their feet remained stationary. Participants performed repetitions for 3 min.

4. Alternating Step-Ups:

From a standing position with feet shoulder-width apart, participants stepped onto a step with one foot while maintaining balance, then brought the same foot back down and repeated the movement with the other leg. Participants performed repetitions for 5 min.

5. Lunges:

Starting from a standing position with feet shoulder-width apart, participants stepped forward with one leg, bent both knees slightly, lowered their body as far as possible, and then pushed back to return to the starting position with the same leg. This movement was repeated alternately with both legs. The same movement was also performed by stepping backward and laterally, with participants bending their knees and lowering their body before returning to the starting position in each direction. Participants performed repetitions for 5 min.

6. Multi-Path Balance Walk:

Participants walked as steadily as possible, incorporating patterns such as forward and backward walking over several meters, lateral steps to the left and right, and tandem walking, where one foot was placed directly in front of the other with the heel touching the toe. They also navigated small obstacles, turning as necessary. Participants performed these walking patterns for 5 min.
